# Recent Molecular Advances on Downstream Plant Responses to Abiotic Stress

**DOI:** 10.3390/ijms13078628

**Published:** 2012-06-04

**Authors:** Sávio Pinho dos Reis, Aline Medeiros Lima, Cláudia Regina Batista de Souza

**Affiliations:** Institute of Biological Sciences, Federal University of Pará, Guamá, Belém, PA 66075-110, Brazil; E-Mails: saviopr@yahoo.com (S.P.R.); alinemedeiros14@hotmail.com (A.M.L.)

**Keywords:** abiotic stresses, genetic engineering, heat-shock proteins, LEA proteins osmoprotectants, plant response, salinity

## Abstract

Abiotic stresses such as extremes of temperature and pH, high salinity and drought, comprise some of the major factors causing extensive losses to crop production worldwide. Understanding how plants respond and adapt at cellular and molecular levels to continuous environmental changes is a pre-requisite for the generation of resistant or tolerant plants to abiotic stresses. In this review we aimed to present the recent advances on mechanisms of downstream plant responses to abiotic stresses and the use of stress-related genes in the development of genetically engineered crops.

## 1. Introduction

Global effects on desertification, soil salinization, atmospheric CO_2_ enrichment, nutrient imbalances (including mineral toxicities and deficiencies), and effects of other pollutants are predicted to cause dramatic changes in the environment of agricultural lands. In a world where population growth exceeds food supply, researchers should focus efforts to find solutions that may help plants overcome stress caused by increasingly challenging environmental conditions [1,2].

Plants respond and adapt to continuous environmental fluctuations by appropriate physiological, developmental and biochemical changes to cope with these stress conditions. The stress in plants is an induced physiological situation when there is severe or constant change in the environment or when normal conditions are aggressive, altering the physiological and adaptive pattern of plants. As an example of the changes that induce abiotic stress in plants, we can mention the variations of temperature, moisture, aqueous saline, soil pH, radiation, and pollutants, such as heavy metals and mechanical damage [3]. All of these environment modifications produce physiological reactions in their cells of genetic origin [4].

These abiotic stress conditions also cause extensive losses to agricultural production worldwide, because they affect negatively plant development and productivity. It is estimated that less than 10% of the world’s arable lands may be free of major environmental stresses. Up to 45% of the world’s agricultural lands are subject to continuous or frequent drought and 19.5% of irrigated agricultural lands are considered saline [2]. Also, crops and other plants are routinely subjected to a combination of different abiotic stresses [5]. In drought areas, for example, many crops encounter a combination of drought and other stresses, such as heat or salinity. Together, these environmental stresses reduce the average yields for major crop plants by 50% to 70%. Individually, stress conditions such as drought, salinity, heat or cold have been the subject of intense research [1,5].

To survive under such conditions, plants have evolved intricate mechanisms to perceive external signals, allowing optimal response to environmental conditions. Responses to abiotic stresses occur at all levels of organization. Cellular responses to stress include adjustments of the membrane system, modifications of cell wall architecture, changes in cell cycle and cell division. To this list must also be included the synthesis of specific endogenous and low-molecular-weight molecules that primarily regulate the protective responses of plants against both biotic and abiotic stresses, such as salicylic acid, jasmonic acid, ethylene, and abscisic acid [6]. At the molecular level, this response also includes the expression of stress-inducible genes involved in direct plant protection against stress [7–9]. Transcriptome analysis using microarray [10–12] has revealed that abiotic stress-induced genes could be divided into two major groups according to the functions of their products. The first group consists of a large number of proteins: enzymatic and structural proteins, such as membrane proteins, enzymes for osmolyte biosynthesis, detoxification enzymes (glutathione S-transferases, hydrolases, superoxide dismutases and ascorbate peroxidases) and other proteins for macromolecular protection (such as LEA protein, chaperons and mRNA binding protein) [13]. The second group comprises a variety of regulatory proteins (such as transcription factors, protein kinases, receptor protein kinases, ribosomal-protein kinases and transcription-regulation protein kinase, *etc*.) and signal transduction proteinases (phosphoesterases and phospholipase C, *etc*.) involved in the regulation of cascades of gene expression [13].

Furthermore, plant acclimation to a particular abiotic stress condition requires a specific response that is linked to the precise environmental conditions that the plant encounters. Thus, molecular, biochemical and physiological processes set in motion by a specific stress condition might differ from those activated by a slightly different composition of environmental parameters. Transcriptome profiling studies of plants subjected to different abiotic stress conditions showed that each different stress condition tested generates a somewhat unique response, and little overlap in transcript expression could be found between the responses of plants to abiotic stress conditions such as heat, drought, cold, salt, high light or mechanical stress. Each abiotic stress condition requires a unique acclimation response, anchored to the specific needs of the plant, and that a combination of two or more different stresses might require a response that is also unique [14,15].

Therefore, the development of genetically engineered plants by the introduction and/or overexpression of selected genes, such as the silencing of specific genes, seems to be a viable option to the breeding of resistant plants [3]. Genetic engineering would be a faster way to insert beneficial genes than through conventional breeding too. Also, it would be the only option when genes of interest originate from cross barrier species, distant relatives, or from non-plant sources. Indeed, there are several genes whose correlative association with resistance has been tested in transgenic plants. Following these arguments, several transgenic approaches have been used to improve stress tolerance in plants [3]. Here, our goal was to show the main functional genes in plants in response to the most common abiotic stresses (drought, salinity, cold, heat and mechanical damage), and their biological roles in the improvement of plants to these stresses.

## 2. Heat-Shock Proteins

Temperatures above the normal optimum cause heat stress at different levels in all living organisms. Heat stress, often associated with salinity and drought stress, disturbs cellular homeostasis, and causes denaturation and dysfunction in many proteins, leading to severe retardation in growth, development and even death. Worldwide, extensive agricultural losses are attributed to heat, often in combination with drought or other stresses. The synthesis and accumulation of heat shock proteins (Hsps) are assumed to play a central role in the heat stress response and in tolerance to high temperatures in all plants, and other organisms [16,17].

The heat stress response is a highly conserved reaction caused by exposure of an organism tissue or cells to sudden high temperature stress, and it is characterized by rapid induction and transient expression of Hsps. As many molecular chaperones are stress proteins and many of them were originally identified as heat-shock proteins, the names of these molecular chaperones follow their early nomenclatures and are referred here as Hsps [18]. So, these proteins primarily function as molecular chaperones to control the proper folding and conformation of both structural (*i.e.*, cell membrane) and functional (*i.e.*, enzyme) proteins, ensuring the correct function of many cellular proteins under conditions of elevated temperature. The primary protein structure for Hsps is well conserved in organisms ranging from bacteria and other prokaryotes to eukaryotes such as higher animals and plants. This conservation ensures a close involvement in the protection of the organism against heat shock and the maintenance of homeostasis [16,19,20].

Hsps are located in both the cytoplasm and organelles, such as the nucleus, mitochondria, chloroplasts and endoplasmatic reticulum. Five major families of Hsps are conservatively recognized, with some of them designated by their approximate molecular weights: the Hsp70 (DnaK) family; the chaperonins (GroEL and Hsp60); the Hsp90 family; the Hsp100 (Clp) family; and the small Hsp (sHsp) family, with proteins with molecular weight between 15 and 42 kDa. The best known families are the chaperonins and the Hsp70 family [18,21,22].

The transcription of Hsp encoding genes is controlled by regulatory proteins called heat stress transcription factors (Hsfs), which exist as inactive proteins mostly found in the cytoplasm [23]. Thus, the heat stress response is controlled by Hsfs, which are activated by the presence of Hsps, acting by binding to the highly conserved heat shock elements in the promoters of target genes (Hsps), activating expression of them [2]. Heat shock transcription factors and the promoter heat shock elements are among the most highly conserved transcriptional regulatory elements in nature [24]. In addition to mediating a relatively large part of the defense response of eukaryotes to heat stress, Hsfs are also thought to be involved in different pathological conditions, cellular responses to oxidative stress, heavy metals, amino acid analogs and metabolic inhibitors, and certain developmental and differentiation processes [20,24,25].

Several studies have been reported about the role of Hsfs in heat stress. In the tomato, HsfA2 was up-regulated early in development [26]. HsfA2 also improved heat and osmotic tolerance in wild *Arabidopsis* [27]. Also in *Arabidopsis*, Hsfs are induced by all major abiotic stresses: heat, cold, osmosis and salt [28]. There are reports about Hsfs regulating other Hsfs as well. HsfA1d and HsfA1e are key regulators of HsfA2 in *Arabidopsis* under heat and high light stress [29]. Also, attempts to increase thermotolerance by overexpression of a single Hsf or Hsp gene have had limited impact because of the genetic complexity of the heat stress response [16]. Hahn *et al*. [30] found a versatile regulatory mechanism in the tomato, where Hsp70 and Hsp90, together regulate different Hsfs. Some reports also showed a role for Hsfs under other abiotic stresses. HsfA2 enhances the anoxia tolerance in wild *Arabidopsis*, besides the heat tolerance [31]. HsfA2 also is induced under salt and drought stress, as reported in rice [32]. The main studies on plant genetic transformation with Hsp genes have investigated mostly heat stress and thermo-tolerance. Positive correlations between the expression levels of several Hsps and stress tolerance have been described extensively by functional genomics and proteomics in different plant species. Comparison of expression data under variable conditions, for example from different tissue types, developmental stages, growth conditions, or applications and durations of stress treatments, shows related patterns of transcript accumulation, with the expression of about 2% of the genome being affected [16,17]. In the tomato, for instance, several Hsps are induced by heat stress [33]. Nine Hsps were reported as up-regulated in rice under heat stress [34]. In *Porphyra seriata*, PsHsp70 enhanced heat stress tolerance [35]. Sung & Guy [36] also related an altered expression in these proteins in *Arabidopsis*. They noticed an increased expression of some Hsp70 under heat stress. Overexpression of Hsp101 from *Arabidopsis* in rice plants, that are sensitive to heat stress, resulted in a significant improvement of growth performance during their recovery [37]. sHsps were also up-regulated under heat treatment in *Arabidopsis* [27]. Hsp17-CII is activated early in the development under heat stress [26]. Hsp90 was greatly induced by heat stress, but not by other abiotic stresses [27]. In a proteomic analysis, Neilson *et al*. [38] found various Hsps up-regulated by heat stress in many plant species.

However, heat is not the only stress treatment that leads to elevated expression of many Hsps. Some are also induced by cold, salt, drought and other non-heat stresses [22]. A study with *Arabidopsis* revealed there are some Hsps induced by different stresses, with changes in expression under a number of environmental conditions [28]. Also, an enhanced salt tolerance was obtained in *Escherichia coli* transformed with the cytosolic chaperonin CCP-1a from *Bruguiera sexangula*, resulting in a significant osmoprotective effect [39]. Salt stress induced the expression of six different Hsps in rice [34]. Hsp100 was up-regulated under salt and drought stress, but not under heat stress [27]. In rice, there was an increased expression of Hsp70 under different kinds of stress [40]. Overexpression of Hsp17.6A improves heat and osmotic tolerance in *Arabidopsis* [27]. In *Ginkgo biloba*, all three GbHSPs of the study were up-regulated under cold stress, whereas extreme heat stress only caused up-regulation of one of them [41]. Heavy metal stress tolerance was also reported. For instance, Hsp90.3 was up-regulated in *Arabidopsis* under cadmium and arsenic stresses [42]. Sometimes a second abiotic stress, when combined with heat stress, increases the Hsp expression. In wheat, the highest Hsp expression was established under the combined drought and heat stress [43]. These results corroborated the hypothesis of Mitler (2006) that simultaneous exposure to different abiotic stresses results in the activation of several stress response pathways. On the other way, some Hsps can be down-regulated under specific stress, such as two Hsps from rice that were down-regulated under cold stress [34].

In conclusion, these data demonstrate divergent functions of Hsp and Hsf genes in response to distinct abiotic stresses. It is clear that the main role of the majority of Hsps/Hsfs is to increase heat tolerance. But there are a number of proteins in plants involved in many other abiotic stresses, individually or combined, that help plants respond to different degrees of environmental changes.

## 3. Osmoprotectants

High soil salinity is one of the important environmental factors that limits distribution and productivity of major crops, causing yield losses. It reduces the ability of plants to take up water, thus leading to reduction in growth rate, due to a hormonal signal generated by the roots [44]. Although salinity is a major problem for agriculture in arid and semi-arid regions of the world, this environmental adversary can be observed in other areas as well, since it affects approximately 40% of irrigated land to various degrees [45,46].

However, to combat this problem, a major strategy for the utilization of salt-affected lands is to grow salt-tolerant crops/cultivars on such soils. This strategy is considered the most efficient and economical means of utilizing salt-affected soils and hence a permanent solution to the problem. The most efficient way for this is through a transgenic approach [46]. As different stress-regulated genes have roles in salt tolerance, transgenic research promotes the alteration of expression levels of native genes or by incorporating exogenous genes for a desired trait [44].

Salt stress results in a wide variety of physiological and biochemical changes in plants, such as the activation of salt-inducible genes, such as transcription factors [47], bZIP [48], LEA [49], RING zinc-finger [50] and the large scale production and accumulation of osmolytes. Plants accumulate the derivatives of these low molecular weight solutes to mitigate the detrimental effects of salt stress by lowering the water potential of cells or by protecting various cellular structures and proteins during stress. The accumulation of compatible osmolytes involved in osmoregulation allows additional water to be taken from the environment [51,52]. On the basis of this understanding, enzymes that catalyze steps in the biosynthesis of these compatible osmolytes are considered to be examples of salt-stress-tolerance effectors [44,45].

Here, we will outline recent achievements in the generation of transgenic plants with modified molecular salt/osmotic stress responses. This has been attained through the expression of gene encoding enzymes that catalyze production of most common osmolytes: proline, glycine-betaine, trehalose, and sugar alcohols such as mannitol and sorbitol.

### 3.1. Proline

Proline is an amino acid known to occur widely in higher plants and in response to environmental stresses (especially salt/osmotic stress), and normally accumulates in large quantities. Under salt/osmotic conditions, it contributes to the stabilization of proteins, membranes and subcellular structures in cytosol, and protecting cellular functions by scavenging reactive oxygen species. Furthermore, it is known to induce expression of salt stress responsive genes, which possess proline-responsive elements [51,53]. Accumulation of proline could be due to *de novo* synthesis or decreased degradation, or both, and it is synthesized from glutamate and ornithine. In plants, the main pathway is from glutamate, which is converted to proline by two successive reductions catalyzed by pyrroline-5-carboxylate synthases (P5CS) and pyrroline-5-carboxylate reductases (P5CR), respectively. Ornithine is the alternative precursor for proline, which can be transaminated to P5C by Orn-d-aminotransferase (OAT), a mitochondrial located enzyme [51,54].

In recent years, several attempts were made to increase the level of proline accumulation in plants by transferring the genes associated with the biosynthetic pathway. Proline-level increase by overexpressing P5C enzymes were observed in several studies and in different plant species. In tobacco, the increase in expression of GK74 and GPR promoted the overproduction of proline [55]. The activities of P5CS and P5CR were significantly enhanced in the leaves of *Morus alba*s with decreasing leaf water potentials [56]. In cactus pear, salt stress increased the expression of P5CS and induces proline accumulation [57]. On the other hand, proline levels decreased when P5C enzymes are silenced. A P5CS in *Arabidopsis* was knocked-out, reducing the proline synthesis [58].

The down-regulation of proline dehydrogenase (ProDH) also increases proline levels. The transcription of antisensing ProDH improved the production of proline in tobacco [55]. In the mulberry, the activities of proline dehydrogenase were reduced with progressive increase in water stress [56]. Also, there are studies about the proline alternative pathway. The activities of ornithine transaminase were increased in *Morus alba* with low leaf water potentials [56]. In cashew plants, it was reported that under salt-induced stress the levels of ornithine were increased [59]. In *Hibiscus tiliaceus* a contradictory result was reported. Formate dehydrogenase, negatively associated with the accumulation of proline in response to osmotic stress, was found highly up-regulated during salt stress [60].

### 3.2. Glycine Betaine

Glycine betaine (GB) is a fully *N*-methyl-substituted quaternary ammonium derivative of glycine that is found in bacteria, hemophilic archaebacteria, marine invertebrates, mammals and plants [53,61]. Among the many compounds known in plants, GB is accumulated at high levels in response to abiotic stress, mainly to salt/osmotic stress. Levels of accumulated GB are generally correlated with the extent of stress tolerance, and vary considerably among plant species and organs [62]. GB is abundant mainly in chloroplast where it plays a vital role in the adjustment and protection of the thylakoid membrane, thereby maintaining photosynthetic efficiency and its highly ordered state at non-physiological salt concentrations via ROS scavenging. Also, at lower concentrations, GB effectively stabilizes the quaternary structures of enzymes and complex proteins [62,63].

In higher plants, GB is synthesized and accumulated in chloroplasts from serine via ethanolamine, choline, and betaine aldehyde. Choline is converted to GB through a two-step pathway: first to betaine aldehyde, by choline monooxygenase or an enzyme with similar function, which is then converted to GB by betaine aldehyde dehydrogenase (BADH). Although other pathways such as direct N-methylation of glycine is also known, the pathway from choline to glycine betaine has been identified in all GB-accumulating plant species [53,64].

Many researches corroborate the hypothesis that GB plays an important role in tolerance to abiotic stress, showing natural accumulators such as spinach, maize, sugar beet, and barley, that synthesize GB during exposure to salt, drought, and low temperature stresses [64]. Furthermore, both the exogenous application of GB and the introduction of the GB-biosynthetic pathway genes to create lines of GB-accumulating transgenic plants increased the tolerance of such plants to osmotic stress [61,62].

There are recent advances in salt tolerance via exogenous introduction of GB. In tobacco, accumulation of glycine betaine provides a protection against salt-induced stress [65]. In the tomato, the application of exogenous GB also improved salt tolerance [66], as well as in *Atriplex halimus* [67], papaya [68], maize [69] and soybean [70]. Also, GB introduction confers tolerance to other abiotic stress: cadmium stress resistance has been reported in cultured tobacco cells [71], and high temperature stress tolerance in the tomato [72]. On the other hand, an exogenous application of GB in the sunflower was not found to be effective in reducing the negative effects of drought stress [73].

With increasing knowledge of genomics and proteomics coupled with gene engineering technologies, several plant species have been engineered with genes of the GB biosynthetic pathway in order to generate plants with tolerance to osmotic stress. The heterologous expression of a choline dehydrogenase, an important enzyme in the GB synthesis pathway, improved salt tolerance in tobacco [74]. He *et al*. [75] reported similar results by the introduction into wheat of the betA gene, a choline dehydrogenase from *Escherichia coli*. A year later, the same research group also showed an improvement in drought resistance and biomass in wheat seedlings with increased glycine betaine [76]. In cotton, salt stress resistance and improved seed yield were obtained by transformation of the same betA gene [77]. Tobacco plants with a transgenic BADH gene showed similar results [78,79]. GB-accumulating transgenic plants also showed improved tolerance to other abiotic stresses, such as drought, heat [80] and cold stress [81]: all reported in wheat.

Some studies also have showed that the introduction of more than one transgene improves the tolerance to osmotic stress. Duan *et al*. [74] has reported that tobacco plants, with the expression of two genes related to GB biosynthesis, are more tolerant than lines with a single transgene. In rice, lines expressing codA gene, that converts choline into GB in a single step, in association with several other stress responsive genes, were water-stress tolerants [82].

### 3.3. Trehalose

Trehalose, a non-reducing disaccharide sugar, is a compatible solute composed of two molecules of glucose bound by an α, α (1-1) linkage. It is well known as an abiotic stress protectant in a wide range of organisms, including bacteria, fungi, invertebrates and plants, working in the stabilization of membranes and lipid assemblies and in the stabilization of biological macromolecules in the folded state, forming a molecular complex [63,83,84]. These protective properties make trehalose an ideal stress protectant [83]. In plants, its biosynthesis is catalyzed by two key enzymes: trehalose-6-phosphate synthase (TPS) and trehalose- 6-phosphate phosphatase (TPP) [84,85]. It is not thought to accumulate to detectable levels in most plants, with the exception of the desiccation-tolerant “resurrection plants”. Indeed, for years trehalose in plants was thought to be restricted to these resurrection plants. Later, it was detected in other plants with the application of inductors, such as validamycin A, an inhibitor of trehalase, the trehalose degrading enzyme [86].

There were significant advances in osmotic stress tolerance with the trehalose genetic engineering approach. In rice, the overexpression of TPP, the enzyme responsible for one step in the trehalose pathway from glucose, conferred abiotic stress tolerance [87]. In the same crop, Li *et al*. [85] and Redillas *et al*. [88] also enhanced abiotic stress tolerance by expression of TPS. Under drought stress, transformed maize plants with high levels of trehalose improved their resistance and their biomass when compared to wild lines [89]. Even a low expression of trehalose-related genes can show significant results. In the potato, the observed expression pattern of a TPS from *Saccharomyces cerevisiae* was weak, but was sufficient to alter the drought response of plants [90]. Furthermore, multiple transgenes have strongly enhanced the response to abiotic stress. Suárez *et al*. [91] reported a study with alfalfa, where yeast TPP and TPS genes were fused and expressed. As a result, transgenic plants displayed a significant increase in drought, freezing, salt, and heat tolerance. Induction of trehalose in symbiont organisms of some plants has also increased the resistance of plants to abiotic stress. In *Bradyrhizobium japonicum*, the root nodule symbiont bacteria of soybeans, trehalose accumulation due to different trehalose biosynthetic genes enhanced survival under conditions of salinity stress and also played a role in the development of nitrogen-fixing root nodules [92].

The exogenous application or induction of trehalose without genetic engineering to obtain salt/osmotic tolerance is also common. Li *et al*. [93] has reported thermal tolerance with the addition of trehalose in wine yeast. The induction with validamycin A improved the salt tolerance of *Medicago truncatula* [94]. Exogenous trehalose recovered rice seedlings from salt stress [95]. Finally, trehalose treatment reduced the adverse effects of salinity stress on the metabolic activity of maize seedlings [96].

### 3.4. Other Sugar Alcohols

Mannitol is a widely distributed sugar alcohol found in many plants and other organisms. It is synthesized in mature leaves from mannose- 6-phosphate, through the combined action of a NADPH-dependent mannose-6-phosphate reductase (M6PR) and a mannitol-6-phosphate phosphatase. It is transported to sink tissues where it can be either stored or oxidized to mannose by an NAD-dependent mannitol-1phosphate dehydrogenase (mtlD) [97,98]. In addition to its role as a primary photosynthetic product and translocated carbohydrate, in many species mannitol production may confer several potential advantages, functioning in osmotic and salt stress tolerance by serving, when accumulated, as a compatible solute or osmoprotectant [99].

Plants transgenic for mannitol-related genes have shown increases in stress tolerance, particularly salt tolerance. Additional evidence for a role for mannitol in salinity tolerance was obtained when *Nicotiana tabacum*, *Populus tomentosa* and other plants were genetically engineered through introduction of mtlD, resulting in more salt-tolerant plants [27,98]. The overexpression of mtlD resulted in the accumulation of a small amount of mannitol and also in the improved tolerance to salinity and drought in *Arabidopsis* and tobacco [63]. In *Pinus taeda*, salt tolerance assays demonstrated that transgenic plants with increased expression of mtlD had an increased ability to tolerate high salinity [99]. In wheat, the induced expression of the mtlD gene for the biosynthesis of mannitol in wheat improves tolerance to water stress and salinity [100]. Salt and drought significantly increased mannitol transport activity and the expression of a mannitol dehydrogenase gene in *Olea europaea* [98]. In an alternative approach, plants can be transformed with the M6PR gene. *Arabidopsis* plants transformed with the M6PR gene from celery were dramatically more salt tolerant as exhibited by reduced salt injury, less inhibition of vegetative growth, and increased seed production relative to the wild type. In this study, the M6PR gene induced the expression of a variety of other stress-inducible genes [101].

Sorbitol, a six-carbon sugar alcohol and another important representative of osmoprotectants, is a primary photosynthate, a major translocated form of sugars and the preferential storage carbohydrate of fruit trees, mainly woody members of the Rosaceae family [102–104]. Sorbitol may be involved in providing tolerances against abiotic and biotic stresses. It plays a key role in osmotic adjustment under drought, cold and salinity conditions. Its synthesis shares a common hexose phosphate pool with sucrose production in cytosol. In source leaves, glucose-6-phosphate is converted to sorbitol-6-phosphate by sorbitol-6-phosphate dehydrogenase (S6PDH). Afterward, sorbitol-6-phosphate is used to form sorbitol via sorbitol-6-phosphatase by dephosphorylation. The reaction catalyzed by S6PDH is the key regulatory step in sorbitol synthesis [105,106].

A common approach to accumulate sorbitol is to submit the plant to stress conditions that induce sorbitol transporter genes. Transporters have been identified in various plants, such as sour berry, apple and *Arabidopsis* [107,108]. It has been shown that different sugar transporters are up-regulated or down-regulated by salt and drought stress [109]. Assessment of sorbitol concentrations in *Plantago major* leaves revealed an accumulation of sorbitol within salt-stressed plants [110]. Analysis of sorbitol levels via high performance liquid chromatography (HPLC) showed that its concentration was elevated in roots, phloem tissues and the leaves of apple plants after the up-regulation of three sorbitol transporter under conditions of osmotic stress in apple plants, enhancing drought tolerance in vegetative tissues [104].

## 4. LEA Protein

Late embryogenesis abundant (LEA) proteins were first described almost 30 years ago in cotton seeds, where they are specifically produced and accumulated during late embryo development [111]. LEA protein expression correlates closely with the acquisition of tolerance against drought, freezing, and salinity stresses in many plants [112–118].

The term “late embryogenesis abundant” has been used for more than 20 years to describe genes/proteins. However, numerous LEA genes were found to be not expressed during seed development and expressed only in vegetative tissues. In addition, considering the animal LEA proteins, the term “late embryogenesis abundant” cannot be applied to all situations. Dure *et al*. [119] suggested a substitute term “water stress protein”, which is not acknowledged widely, possibly because it refers to proteins induced only during water stress. In the past decade, LEA proteins were also found in animals [120], including nematodes [121–125].

LEA proteins are mainly small molecular weight proteins ranging from 10 to 30 kDa [126]; they have been grouped into various LEA families based on the occurrence of amino acid motifs [117,119,127] and can confer protection against different stress conditions; in general, groups 1, 2 and 3 are considered as the three major groups containing most members of the protein family. Group 1 LEA proteins (Pfam 00477) are mostly present in plants and they contain at least one copy of a 20 amino acid motif. For example, group 1 LEA protein of wheat confers tolerance to osmotic stress in yeast [128]. Group 2 LEA proteins or dehydrins (Pfam 00257) are also found in algae and share a common K-segment present in one or several copies; many dehydrins also contain an S-segment (polyserine stretch) that can undergo extensive phosphorylation and a Y-domain (DEYGNP), similar to the nucleotide-binding site of plant and bacterial chaperones. Group 3 LEA proteins (Pfam 02987), also found in nematodes and prokaryotes, contain at least one copy of a 11 amino acid motif. Also, the group 3 LEA protein of rice has been found to induce resistance to drought when over-expressed transgenically [129].

Studies have shown the great potential of LEA in controlling stress as reported by several authors. López *et al*. [130] have studied several winter wheat cultivars for the evaluation on dehydrin accumulation during the exposure of drought stress. Some of them expressed a dehydrin after stress treatment while no dehydrins were detected in non-stress control plants. The presence of dehydrins was related to the acquisition of drought tolerance. Transgenic wheat and oat expressing HVA1, an LEA protein, showed increased desiccation tolerance, biomass productivity, and water use efficiency under high salt, osmotic, or drought conditions via membrane protection [131–133]. The accumulation of wheat WCS19, a cold regulated chloroplast LEA III protein, in transgenic *Arabidopsis* increased resistance to freezing stress, which suggested that WCS19 proteins enhanced freezing tolerance [134]. Studies with cassava (*Manihot esculenta* Crantz) performed by Costa *et al*. [49] revealed that levels of MeLEA3 transcripts were increased under *in vitro* salt stress treatment, indicating its potential role in stress response.

In transgenic rice expressing wheat PMA1595 (LEA I) or PMA80 (LEA II), the accumulation of PMA1595 or PMA80 proteins was associated with increased salt and drought stress tolerance. Accumulation of *Arabidopsis* AtRAB28 (LEA V) protein through transgenic approach improved the germination rate under standard conditions or salt and osmotic stress [135]. The accumulation of the citrus dehydrin CuCOR19 in tobacco conferred less electrolyte leakage than control plants under freezing temperature. Transgenic plants also showed earlier germination and better seedling growth than control plants with chilling treatment (Hara *et al*., 2003). Wheat WCor410 (LEA II) gene introduced into strawberry improved chilling tolerance after previous acclimation treatment [136].

Rapeseed (*Brassica napus*) LEA III gene MEleaN4 introduced into Chinese cabbage (*Brassica campestris*) or lettuce (*Lactuca sativa L*.) resulted in improved drought tolerance [137,138]. The constitutive expression of maize dehydrin rab17 gene in transgenic *Arabidopsis* increased the sugar and proline contents; in addition, these plants showed more tolerance to high salinity conditions and recovered faster from mannitol treatment than non-transformed control plants [139].

## 5. Conclusions and Perspectives

Worldwide, the several kinds of abiotic stresses are major causes of plant diseases and death. Genetic engineering is a viable solution to these problems. Important findings as the role of several Hsps, osmoprotectants and LEA proteins help the construction of transgenic agricultural crops, such as soybean, a widespread transgenic crop in the Americas. As in 2010, USA, Brazil and Argentina have the greatest transgenic crop areas of the world [140]. However, it is important to verify how the tolerance to specific abiotic stress is assessed, and whether the achieved tolerance compares to existing tolerance. The biological effects of changes in the production of different proteins and their effect on yield should be properly evaluated. Also, an approach combining the molecular, physiological and metabolic aspects of abiotic stress tolerance is necessary to acquire a better knowledge of gene expression, as well as the whole plant phenotype assessment under stress. Finally, the future of abiotic stress research should focus on targeting multiple gene regulation, because the change on a single gene expression may not have enough results on the field in question, since genetic regulation is complex. Working with a number of candidate genes can improve the tolerance to different abiotic stresses.
